# Metabolites Associated with Polygenic Risk of Breast Cancer

**DOI:** 10.3390/metabo14060295

**Published:** 2024-05-23

**Authors:** Elizabeth Samuels, Jaclyn Parks, Jessica Chu, Treena McDonald, John Spinelli, Rachel A. Murphy, Parveen Bhatti

**Affiliations:** 1 Cancer Control Research, BC Cancer Research Institute, Vancouver, BC V5Z 1L3, Canada; 2 School of Population and Public Health, University of British Columbia, Vancouver, BC V6T 1Z3, Canada

**Keywords:** polygenic risk score, metabolomics, single-nucleotide polymorphisms, lipoproteins, breast cancer

## Abstract

While hundreds of germline genetic variants have been associated with breast cancer risk, the mechanisms underlying the impacts of most of these variants on breast cancer remain uncertain. Metabolomics may offer valuable insights into the mechanisms underlying genetic risks of breast cancer. Among 143 cancer-free female participants, we used linear regression analyses to explore associations between the genetic risk of breast cancer, as determined by a previously developed polygenic risk score (PRS) that included 266 single-nucleotide polymorphisms (SNPs), and 223 measures of metabolites obtained from blood samples using nuclear magnetic resonance (NMR). A false discovery rate of 10% was applied to account for multiple comparisons. PRS was statistically significantly associated with 45 metabolite measures. These were primarily measures of very low-density lipoproteins (VLDLs) and high-density lipoproteins (HDLs), including triglycerides, cholesterol, and phospholipids. For example, the strongest effect was observed with the percent ratio of medium VLDL triglycerides to total lipids (0.53 unit increase in mean-standardized ln-transformed percent ratio per unit increase in PRS; q = 0.1). While larger-scale studies are needed to confirm these results, this exploratory study presents biologically plausible findings that are consistent with previously reported associations between lipids and breast cancer risk. If confirmed, these lipids could be targeted for lifestyle and pharmaceutical interventions among women at increased genetic risk of breast cancer.

## 1. Introduction

Genome-wide association studies (GWASs) have identified hundreds of germline genetic variants, mostly single-nucleotide polymorphisms (SNPs), as risk factors for breast cancer [1,2,3]. However, most of the identified SNPs have small individual effect sizes, making it difficult to ascribe any clinical significance to an individual carrying a particular SNP [1]. To address the issue of small effect sizes and improve clinical utility, researchers have incorporated multiple SNPs into polygenic risk scores (PRSs) representing the relative lifetime genetic risk of developing cancer for individuals carrying some or all of these SNPs [4]. Understanding the molecular mechanisms through which SNPs contributing to a PRS confer increased risks of breast cancer could reveal targets for prevention, particularly if multiple breast cancer SNPs act on similar pathways. However, the functions of most SNPs associated with breast cancer remain uncertain [5,6].

Metabolites, small molecules that are substrates, intermediates, and end products of cellular metabolism, may initiate or promote carcinogenesis themselves or may serve as markers for other carcinogenic processes [7,8]. Multiple studies have evaluated pre-diagnostic measures of metabolites in blood in association with breast cancer risk [9,10,11,12,13]. For example, in a large prospective study, acylcarnitine C2 was found to be associated with a 1.2-fold increased risk of breast cancer [11]. Acylcarnitine is implicated in several mechanisms of breast cancer development, including insulin resistance and fatty acid oxidation.

To date, there has been no research evaluating the metabolic profiles of individuals at different levels of genetic risk of developing breast cancer. By conducting an exploratory analysis of genomic and metabolomic data previously generated for a group of 143 women, the objective of this study was to reveal the mechanisms that may underlie polygenic risk of breast cancer.

## 2. Materials and Methods

### 2.1. Study Population

We used data from the B.C. Generations Project (BCGP; data accessed September 2021 to September 2023), the largest population-based health study in British Columbia, Canada [14]. The BCGP is a prospective cohort study of 29,736 participants from across British Columbia who were aged 35–69 when they were recruited between 29 May 2009 and 31 August 2016. At baseline, after providing written informed consent, participants completed a health and lifestyle questionnaire and provided height and weight measurements for the calculation of body mass index (BMI). Buffy coat and serum were extracted from non-fasting blood samples donated at baseline. After processing and aliquoting, samples were stored at −80 °C.

### 2.2. Metabolomics Data

A group of 1320 BCGP participants were included in previous studies of metabolomics and cancer preventive behaviors [15,16]. Individuals were randomly selected from among non-smokers with no history of cancer at baseline and who were not pregnant at the time of blood sample collection. Serum samples were sent to Nightingale Health Ltd. (Vantaa, Finland) where a nuclear magnetic resonance (NMR) platform was used to generate 225 metabolite measures, including concentrations (mmol/L) of various lipids (very small, small, medium, large, and very large subtypes of very low-density lipids (VLDLs), low-density lipoproteins (LDLs), and high-density lipoproteins (HDL, HDL2, HDL3)), fatty acids, fatty acid composition, ketone bodies, fluid-balance-related metabolites, amino acids, glycolysis, and gluconeogenesis-related metabolites [17]. Additional measures included ratios of lipid subtypes, lipid subtypes expressed as percentages of total lipid concentrations, and mean diameters of VLDL, LDL, and HDL particles. Glucose and lactate measures were lower and higher than expected, respectively, indicating the occurrence of glycolysis during sample collection and handling, so these metabolites were excluded [16].

### 2.3. Genomics Data

For 1000 BCGP participants of European ethnicity, genome-wide SNP data were generated on DNA using the Affymetrix Axiom 2.0 UK Biobank gene chip. DNA was extracted using the Qiagen FlexiGene Extraction kit on buffy coats stored at −80 degrees Celsius. Genotyping was completed by the Clinical Genomics Centre at Mount Sinai Hospital in Toronto, ON, Canada. Data were generated for 836,727 SNPs, of which 768,633 passed quality control assessment (call rate ≥ 95%, good allele cluster resolution, and passed the Hardy–Weinberg equilibrium test at a *p* = 10^−6^ threshold). All genotype data from 10 of the participants failed quality control assessment, leaving high-quality data for 990 participants. For these participants, vcf files (one for each chromosome) were created using VcfCooker and submitted to the Michigan Imputation Server [18]. Imputation was based on the HRC r1.1 2016 reference panel. Uploaded variants were excluded from the process by the Michigan Imputation Server if they included invalid or mismatched alleles, were duplicates, and/or were monomorphic sites. A total of 702,383 SNPs for each participant were utilized by the server, which imputed a total of 39,609,539 SNPs.

### 2.4. Polygenic Risk Score

The PRS developed by Mavaddat et al. (2019), which consists of 313 genetic variants, among which are 266 SNPs and 47 insertions/deletions, was selected for this study [19]. The PRS was constructed using large training (11,428 breast cancer cases and 18,323 controls) and validation datasets (UK Biobank—3215 breast cancer cases among 190,040 women). The authors compared this PRS to two previously published PRSs and found the new PRS to be significantly more predictive of breast cancer risk on the basis of a stronger hazard ratio and a more optimal area under the curve [19,20]. The authors also conducted goodness-of-fit tests and evaluated the performance of the PRS at the tails of the distribution and found no deviation from the model, an important characteristic that they noted was not met by many other PRSs.

Since we lacked data on the 47 insertions/deletions in the Mavaddat et al. (2019) PRS, we focused our evaluation on the 266 SNPs included in the PRS. The PRS was derived using a weighted coefficient, multiplied by 0, 1, or 2 based on the number of copies of each specific SNP. These were then added together to compute a numeric score estimating the lifetime genetic risk of developing breast cancer for each participant.

### 2.5. Statistical Analysis

Statistical analyses were completed using R version 4.0.3. Fifty-one of the metabolite measures were found to have at least one measure below the limit of detection (LOD), though no single metabolite had more than 12% of measures below the LOD. Values below the LOD were set to half of the minimum measurement of that metabolite in the study population [16]. Histograms of the 223 metabolite measures revealed that most were right-skewed and so all measures were ln-transformed. Measures were also mean-standardized to enhance the comparability of results across the varying metabolite measures:

z = (ln-transformed measurement-overall mean of ln-transformed measurements)/standard deviation of ln-transformed measurements

For each of the 223 z-standardized ln-transformed metabolite measures, a linear regression analysis was conducted to evaluate associations with the PRS. To address the issue of multiple comparisons, the Benjamini and Hochberg approach was applied to set the false discovery rate (FDR) at 10% [21]. For each association, a q-value was generated, and any association with q ≤ 0.1 was deemed statistically significant.

## 3. Results

**Figure 1:**
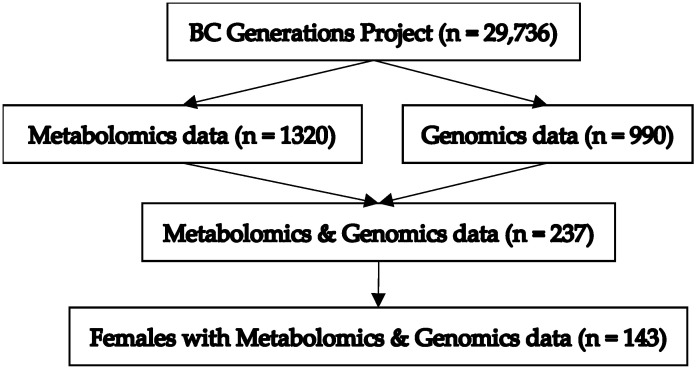
Depiction of inclusions/exclusions producing final study sample size.

A total of 237 (biological sex: 94 males, 143 females) BCGP participants had both genomic and metabolomic data. The current study was restricted to the 143 females in this group given the focus on breast cancer. See Figure 1 for a flowchart depicting inclusions/exclusions that produced the final sample size. Characteristics of these participants are summarized in Table 1. The mean (standard deviation; SD) age of participants at the time of blood collection was 55.7 years (7.7). The mean (SD) BMI of participants was 26.0 kg/m^2^ (4.8). Only 11 (7.7%) participants reported no alcohol consumption. The mean (SD) age of first menarche was 13.0 (1.4). The mean (SD) gravidity was 2.5 (1.6). A total of 17 of the 143 participants reported that their mother had been previously diagnosed with breast cancer, and 6 participants reported that at least one sibling had been previously diagnosed with breast cancer. The mean (SD) PRS was 0.051 (0.51), ranging from −1.36 to 1.52.

PRS was found to be statistically significantly associated with 45 of the 223 metabolite measures (q ≤ 0.1) Each of these 45 measures are listed along with β-estimates, *p*-values, and q-values in Table 2. The results for all 223 metabolite measures are provided in the Supplementary Materials.

A total of 29 of the 45 measures were related to VLDL, of which 27 were positively associated with PRS. For example, the strongest effect was observed with the percent ratio of medium VLDL triglycerides to total lipids (0.53 unit increase in mean-standardized ln-transformed percent ratio per unit increase in PRS). PRS was significantly negatively associated with two VLDL measures, including percent ratio of medium VLDL cholesterol esters to total lipids (0.41 unit decrease in mean-standardized ln-transformed percent ratio per unit increase in PRS) and percent ratio of medium VLDL total cholesterol to total lipids (0.39 unit decrease in mean-standardized ln-transformed percent ratio per unit increase in PRS). Thirteen of the forty-five significant measures were related to HDL, of which eight were negatively associated with PRS. For example, the strongest negative effect among HDL measures was observed for mean diameter of HDL particles (0.37 unit decrease in mean-standardized ln-transformed particle diameter per unit increase in PRS). Additional measures associated with PRS that were not VLDL- or HDL-specific included the ratio of apolipoprotein B to apolipoprotein A1 (0.37 unit increase in mean-standardized ln-transformed ratio per unit increase in PRS), the ratio of triglycerides to phosphoglycerides (0.36 unit increase in mean-standardized ln-transformed ratio per unit increase in PRS), and serum triglyceride concentration (0.37 unit increase in mean-standardized ln-transformed concentration per unit increase in PRS).

## 4. Discussion

In evaluating the relationship between a breast cancer PRS and metabolites, measured by the Nightingale platform, various measures of VLDL and HDL, including triglycerides, cholesterol, and phospholipids, were significantly associated with PRS. Most of the VLDL measures were positively associated with PRS, while most of the HDL measures were negatively associated with PRS. Overall, the findings suggest that changes to these lipids may contribute to the mechanisms underpinning the polygenic risk of breast cancer.

There has been conflicting evidence for the association between lipids and breast cancer risk, though a recent meta-analysis of 26 prospective studies found that, overall, HDL cholesterol levels tend to be significantly negatively associated with the risk of breast cancer. While this is consistent with our findings of negative associations between multiple HDL cholesterol measures and PRS, the meta-analysis did not observe significant associations with triglycerides, apolipoprotein A, and LDL cholesterol [22]. It has been hypothesized that the primary mechanism by which HDL lowers breast cancer risk is through its role in anti-inflammatory pathways and the regulation of apoptosis [23].

No prospective studies of VLDL and breast cancer risk were identified, although several case–control studies of breast cancer using post-diagnostic samples have observed associations with breast cancer risk [24,25,26,27]. One case–control study found that serum VLDL and triglyceride levels were significantly higher in breast cancer patients when compared to those with benign breast disease [24]. Another case–control study found elevated VLDL and triglyceride levels among breast cancer patients when compared to normal controls, and that the increase was more pronounced in the later stages of the disease [25]. A case–control study of women with breast cancer found that higher VLDL cholesterol levels were significantly associated with breast cancer [26].

Triglycerides are the major lipid associated with VLDL and are transported by VLDLs to muscle and adipose tissue [28]. One potential mechanism through which triglycerides may impact breast cancer risk involves metabolic disease. High triglyceride levels are associated with metabolic disease, which is, itself, associated with increased breast cancer risk [29]. High levels of triglycerides have also been shown to increase COX-2 expression in mammary arteries [30] and COX-2 overexpression is associated with mammary carcinogenesis [31].

To our knowledge, this is the first study to integrate metabolomic and genomic data for the purpose of identifying the mechanisms underlying increased genetic risk of breast cancer. Our novel approach has the potential to inform interventions that can reduce the burden of breast cancer (e.g., circulating lipid levels could be targeted through nutritional or pharmaceutical interventions). The small study sample size did limit our ability to detect modest associations. However, as a preliminary investigation, the findings of this study have identified potential future candidates for metabolomics investigations and will help to motivate future larger-scale evaluations.

Although there are many factors that can impact metabolites, including BMI, age, and lifestyle factors, it was not possible for these to act as confounding factors in our study as they do not influence genetic variation. While adjustment for fasting status at the time of blood collection [32] may theoretically have improved the precision of our analysis, all but one of the participants reported eating the day of blood sample collection, and so it was not included in the analysis.

Confounding due to population admixture is also possible with study designs evaluating phenotypic associations with genetic variants, although it was less likely to be a factor in this study for several reasons. First, the study population was entirely of European descent, and to the best of our knowledge was not concentrated among specific sub-populations of European ancestry. The population was also geographically focused in the Lower Mainland of British Columbia, Canada, which is not an isolated community and has high rates of in- and out-migration, making it less likely for there to be distinct blocks of shared lineages. Second, instead of evaluating individual genes, this study used a PRS; so, in order to have confounding due to admixture, any given sub-lineage or group of shared ancestry would need to independently have a different PRS from the rest of the population, meaning higher or lower risk across many SNPs used within the PRS. The nature of the PRS being a summative score of many SNPs makes it less likely for this phenomenon to occur, although it is still a potential concern.

The PRS used in this study originally contained 47 insertion/deletion polymorphisms for which we did not have data and had to exclude, meaning that any distinct mechanisms underlying the impacts of these variants could not be identified. PRSs have predominantly been generated for European populations, reflecting issues of bias and equity introduced by the limited diversity of populations used in most GWASs [33]. These issues are also true of the data used in this study, meaning that our findings have limited generalizability to non-European populations. Future research will need to address this issue of inclusivity and utilize genomic data from samples that are more representative of diverse populations.

Although the Nightingale NMR platform included over two hundred metabolites, the primary focus of the platform was on lipids, which may not capture the metabolic pathways most relevant to the genetic risk of breast cancer. Conversely, untargeted mass spectrometry methods can measure thousands of metabolites across a broad range of classes and may be better suited for the discovery of novel mechanisms by which PRS drives breast cancer risk.

Our study found that polygenic risk for breast cancer, calculated using a previously developed PRS, was significantly associated with multiple measures of lipid metabolites. This work provides insights into the mechanistic underpinnings of polygenic risk for breast cancer, motivating future larger-scale and more detailed investigations into these relationships.

## Figures and Tables

**Table 1:** Characteristics of study participants (N = 143).

Characteristic	N (%)	Mean (SD)	Min, Max
**Age (years)**		55.7 (7.7)	40, 69
**BMI (kg/m^2^)**		26.0 (4.8)	18.6, 42.4
18.5–25	75 (52.4%)		
>25	68 (47.6%)		
**Alcohol Consumption**			
Never	11 (7.7%)		
<1/month	23 (16.1%)		
1/month	23 (16.1%)		
2–3/month	15 (10.5%)		
1/week	20 (14.0%)		
2–3/week	25 (17.5%)		
4–5/week	14 (9.8%)		
6–7/week	12 (8.4%)		
**Age of menarche (years)**		13 (1.36)	9, 17
**Gravidity**		2.5 (1.57)	0, 8
**Menopausal status at baseline**			
Pre-menopausal	59 (41.3%)		
Post-menopausal	84 (58.7%)		
**Family History of Any Cancer**			
Yes	98 (69%)		
No	45 (31%)		
**Family History of Breast Cancer**			
Mother	17 (11.9%)		
Sibling	6 (4.2%)		

**Table 2:** Metabolites statistically significantly associated with breast cancer PRS.

Metabolite Measure Notation ^a^	Metabolite Measure Description ^a^	β-Estimate ^b^	*p*-Value	q-Value ^c^
S-VLDL-TG	Small VLDL triglycerides	0.45	0.005	0.1
XL-HDL-TG-%	Very large HDL triglycerides to total lipids ratio, %	0.35	0.005	0.1
L-VLDL-TG	Large VLDL triglycerides	0.51	0.006	0.1
L-VLDL-P	Large VLDL particles	0.50	0.006	0.1
L-VLDL-L	Large VLDL lipids	0.50	0.006	0.1
L-VLDL-PL	Large VLDL phospholipids	0.50	0.006	0.1
L-VLDL-CE	Large VLDL cholesterol esters	0.50	0.006	0.1
M-VLDL-TG	Medium VLDL triglycerides	0.45	0.006	0.1
L-VLDL-C	Large VLDL cholesterol	0.50	0.008	0.1
M-VLDL-P	Medium VLDL particles	0.42	0.008	0.1
M-VLDL-PL	Medium VLDL phospholipids	0.41	0.008	0.1
M-VLDL-L	Medium VLDL lipids	0.41	0.009	0.1
S-VLDL-P	Small VLDL particles	0.40	0.009	0.1
L-HDL-TG-%	Large HDL triglycerides to total lipids ratio, %	0.34	0.009	0.1
M-VLDL-TG-%	Medium VLDL triglycerides to total lipids ratio, %	0.53	0.01	0.1
VLDL-TG	VLDL triglycerides	0.42	0.01	0.1
S-HDL-TG	Small HDL triglycerides	0.42	0.01	0.1
VLDL-D	Mean diameter of VLDL particles	0.40	0.01	0.1
S-VLDL-L	Small VLDL lipids	0.39	0.01	0.1
M-HDL-TG-%	Medium HDL triglycerides to total lipids ratio, %	0.39	0.01	0.1
S-VLDL-PL	Small VLDL phospholipids	0.39	0.01	0.1
M-VLDL-FC	Medium VLDL free cholesterol	0.38	0.01	0.1
ApoB/ApoA1	Apolipoprotein B to apolipoprotein A1 ratio	0.37	0.01	0.1
TG/PG	Ratio of triglycerides to phosphoglycerides	0.36	0.01	0.1
HDL2-C	HDL2 cholesterol	−0.33	0.01	0.1
HDL-C	HDL cholesterol	−0.35	0.01	0.1
HDL-D	Mean diameter of HDL particles	−0.37	0.01	0.1
M-VLDL-CE-%	Medium VLDL cholesterol esters to total lipids ratio, %	−0.41	0.01	0.1
XL-VLDL-L	Very large VLDL lipids	0.41	0.02	0.1
L-VLDL-FC	Large VLDL free cholesterol	0.42	0.02	0.1
XL-VLDL-TG	Very large VLDL triglycerides	0.42	0.02	0.1
S-VLDL-TG-%	Small VLDL triglycerides to total lipids ratio, %	0.42	0.02	0.1
XL-VLDL-P	Very large VLDL particles	0.41	0.02	0.1
S-HDL-TG-%	Small HDL triglycerides to total lipids ratio, %	0.40	0.02	0.1
XL-VLDL-C	Very large VLDL cholesterol	0.39	0.02	0.1
XS-VLDL-TG-%	Very small VLDL triglycerides to total lipids ratio, %	0.38	0.02	0.1
Serum-TG	Serum triglycerides	0.37	0.02	0.1
XS-VLDL-TG	Very small VLDL triglycerides	0.36	0.02	0.1
S-VLDL-FC	Small VLDL free cholesterol	0.36	0.02	0.1
L-HDL-C	Large HDL total cholesterol	−0.31	0.02	0.1
L-HDL-CE	Large HDL cholesterol esters	−0.32	0.02	0.1
L-HDL-P	Large HDL particles	−0.32	0.02	0.1
L-HDL-L	Large HDL lipids	−0.32	0.02	0.1
L-HDL-PL	Large HDL phospholipids	−0.33	0.02	0.1
M-VLDL-C-%	Medium VLDL total cholesterol to total lipids ratio, %	−0.39	0.02	0.1

^a^ Annotation provided by Nightengale Health Ltd. ^b^ Mean-standardized, ln-transformed metabolite concentration per unit increase in polygenic risk score. ^c^ Based on Benjamini–Hochberg procedure.

## Data Availability

All data utilized in this manuscript are available through a data access application to the BC Generations Project (https://bcgpresearch.ca/, accessed on 30 April 2024).

## Supplementary Materials

The following supporting information can be downloaded at https://www.mdpi.com/article/10.3390/metabo14060295/s1: Table S1: Metabolite Associations with PRS.
